# Implementing sports injury prevention programmes during and beyond effectiveness trials: a mixed methodologies study

**DOI:** 10.1136/bmjsem-2025-002931

**Published:** 2026-02-20

**Authors:** Joske Nauta, Jelena Haugg, Rico Németh, Evert Verhagen, Carly D McKay, Femke van Nassau

**Affiliations:** 1 Department of Public and Occupational Health, Amsterdam UMC location Vrije Universiteit Amsterdam, Amsterdam, Netherlands; 2 Health Behaviours & Chronic Diseases, Amsterdam Public Health Research Institute, Amsterdam, Netherlands; 3 Medical & Anti-doping, UEFA, Nyon, Switzerland; 4 Amsterdam Collaboration on Health and Safety in Sports, Department of Public and Occupational Health, Amsterdam Movement Sciences, Amsterdam UMC Location Vrije Universiteit Amsterdam, Amsterdam, Netherlands; 5 University of Bath Department for Health, Bath, UK; 6 Department of Community Health Sciences, University of Calgary, Calgary, Alberta, Canada

**Keywords:** Implementation, Review, Injury, Prevention

## Abstract

There is a gap between the evaluation of injury prevention programmes in controlled trials and their use in real-world practice, and implementation research seeks to bridge this gap by supporting programme uptake beyond research settings. This study explored implementation processes and influencing factors during and after effectiveness trials of injury prevention programmes, and examined strategies used for scale-up following trials. Using a mixed methodologies design, corresponding authors of published injury prevention trials were contacted and invited to complete a survey on implementation activities conducted during their trial and, where applicable, during scale-up. A subsample of respondents also participated in semi-structured interviews. In total, 107 injury prevention studies were identified, of which 39 authors completed the survey and nine took part in interviews. Implementation strategies applied during trials were often underreported in published studies but were identified through the survey, with common strategies including in-person training, staff education, and the provision of supportive materials. Only approximately one-third of the studies resulted in scale-up beyond the trial context. Survey responses and interview data highlighted several facilitators and barriers relevant to both trial implementation and scale-up, such as programme context, coach and athlete motivation, and organisational support. Other factors were phase-specific: trial implementation benefited from structured programme development and close researcher involvement, whereas scale-up was more strongly influenced by limited resources and external momentum, such as public events. These findings suggest that to enhance implementation and scale-up of injury prevention programmes, implementation factors should be considered throughout both the development and evaluation phases. Systematic reporting and assessment of facilitators and barriers during trials and broader scale-up initiatives, along with transparent descriptions of implementation strategies used in effectiveness studies, are essential to improve the translation of research findings into practice.

**WHAT IS ALREADY KNOWN ON THIS TOPIC**

- While many sports injury prevention trials have demonstrated success in a controlled setting, scaling up of these programmes into routine practice has proven to be difficult.

**WHAT THIS STUDY ADDS**

- This study explores which implementation strategies were regularly used during sports injury prevention effectiveness trials and scale-up. Furthermore, the study assesses facilitators and barriers experienced both during the trial and scale-up of the preventive programmes.

**HOW THIS STUDY MIGHT AFFECT RESEARCH, PRACTICE OR POLICY**

- This study highlights the limited documentation around applied implementation strategies in trial publications. For injury prevention programmes to be more effectively scaled up into real-world settings, it is essential to ensure that the implementation strategies during trials are more consistently reported.

## Introduction

 Participation in sports and exercise significantly contributes to a healthy and balanced lifestyle.^1 2^ An active lifestyle does, however, also entail a risk for sports and physical activity-related injuries.^3^ These injuries burden both the athlete and society.^4^ Since 1992, when the ‘sequence of prevention’ was first mentioned by van Mechelen *et al*,^5^ many studies on sports injury prevention programmes have been published.^6^ Yet, these programmes are often only tested within a small sample or under strict conditions (eg, randomised controlled trial), and the translation of the effect beyond the trial is limited.^7^

The challenges faced when translating from controlled trials to real-world practice are caused by the context in which the programme needs to be adopted. This involves the athletes, who have both individual features as well as multiple other factors that are associated with injury risk (eg, the level of play in a particular sport). But the athlete is also guided by a specific trainer, who operates within a specific sports culture, which is regulated by a specific association and takes place in a particular socioeconomic class in a specific country.^8^ For a programme to be effective in the real world, Finch^9^ has suggested that more emphasis must be put on the context and implementation processes for injury prevention programmes.

After a programme is successfully evaluated and implemented in a particular setting, a scaling-up process is necessary to achieve health benefits for the population on a broader scale.^7 10^ This seems particularly difficult to accomplish in health promotion studies.^10^ This difficulty also applies to sports injury prevention, where we know that many programmes are effective, but uptake and adoption in the real world remain low.^10^ Since implementation and scale-up are closely intertwined and may include maintenance and dissemination processes, it is not always possible to distinguish between these concepts.^10^ In our study, we defined the implementation phase as the implementation within an effectiveness trial’s context. We considered scale-up to be the programme’s implementation after the effectiveness trial ended; ‘deliberate efforts to increase the impact of successfully tested health interventions so as to benefit more people and to foster policy and program development on a lasting basis’.^11^

Many theories, models and frameworks have been developed to gain a better understanding of the reasons why some programmes are more successfully implemented than others. They can be categorised based on their aim^12^: the first category is the process models, those aim to describe the process of translating research into practice; examples are the Knowledge-to-Action Framework^13^ and the ‘how-to-implement’ model developed by Grol and Wensing.^14^ The second category includes the theories, models and frameworks that aim to understand what influences implementation outcomes. This group includes determinant frameworks like the Consolidated Framework for Implementation Research (CFIR),^15^ but also classic theories like the Theory of Planned Behaviour^16^ and implementation theories such as the Capability, Opportunity, Motivation and Behaviour theory model.^17^ The last category consists of the evaluation frameworks that can be used to structure a process evaluation. The Reach, Effectiveness, Adoption, Implementation, Maintenance framework^18^ is an example of an evaluation framework that is frequently used in injury prevention research. The number of frameworks to evaluate the scale-up of a programme is not as extensive. In public health research, however, the framework for action of Simmons and Shiffman^19^ is widely used.^20^

Although some studies have described factors associated with implementing sports injury prevention programmes in a specific context,^21 22^ little is known about implementation efforts during a trial and what happens to a programme afterwards. This study aimed to better understand the strategies employed when injury prevention programmes were implemented in the context of effectiveness trials. Furthermore, we assessed the strategies, facilitators and barriers employed for scale-up after the trial ended.

## Methods

### Study design and sample

In this study, we employed mixed methodologies through a modified explanatory sequential design. Quantitative data were collected through surveys and by data extraction from original studies (ie, published manuscripts); qualitative data were collected through surveys and semi-structured interviews. In table 1, we present each data source and the section of the results for which the data were used. We identified studies on sports injury prevention programmes by updating the search of a previous review on programme strategies in sports injury prevention.^6^ We approached the corresponding authors of included studies to participate in a short survey. Within the online survey, we asked respondents if they agreed to participate in a follow-up interview on the scale-up of their intervention. We contacted those respondents through email. Written informed consent was acquired online from all participants. The study was reviewed by the Medical Ethical Review Committee of VUmc (Amsterdam UMC; reference number 2020.145) and was exempt from formal ethical approval.

**Table 1:** Overview of the data source within the study

	Included papers		Survey		Interview
	Quan	Qual	Quan	Qual*	Qual
Strategies for implementation and scale-up	X		X		
Process evaluation outcome measures	X		X		
Facilitators and barriers during implementation		X		X	
Facilitators and barriers during scale-up				X	X

* Qualitative data through open-ended question in the survey.

Qual, qualitative data; Quan, quantitative data.

#### Review update

The basis for this study was the review of Vriend *et al*^6^ on programme strategies in sports injury prevention studies. The search for this review was updated in March 2019 using the same methods described in the original paper.^6^ The updated search yielded 37 new studies that matched the inclusion criteria, resulting in 192 studies on programme strategies in sports injury prevention (a flowchart of the search update is available in online supplemental file 1). Since this study aimed to assess factors associated with the implementation of injury prevention programmes, we included only those studies with an educational component or studies focused on exercise-based injury prevention programmes.

#### Data extraction review

We employed the same data extraction strategy described in the original review.^6^ In addition, we extracted details on strategies that may have impacted the programme’s implementation within the effectiveness trial, including supervision, programme delivery, mode of implementation and implementation activities. Also, data on reported adherence, exposure, programme dose, user satisfaction, programme fidelity, compliance and participant enrolment were extracted. Finally, factors mentioned in the text (either Results or Discussion section) that were explicitly identified or could be interpreted as barriers or facilitators were extracted from the data.

#### The survey

The corresponding author of each included paper was contacted through email, which included a private link to the survey. The corresponding author was considered the key informant of the study and was expected to be able to report on the implementation process during the trial. If an email address was bounced, the research team’s files and the internet were consulted to check for a more recent email address. A reminder email was sent after 2 and 4 weeks. If the corresponding author could not be reached, the contact details of a coauthor were identified and the link was sent to a coauthor. Some authors were the corresponding authors on multiple papers, and those authors would receive one invitation to the survey and be asked to complete the survey for each initiative separately.

The survey was distributed through Survalyzer (Survalyzer AG, Zürich, Switzerland), Survalyzer is in line with the requirements of the General Data Protection Regulation. The survey consisted of two parts (see online supplemental file 2). The first part asked about strategies, process evaluation aspects and facilitators and barriers during the trial, while the second part focused on strategies and facilitators and barriers during scale-up after the trial ended. For the strategies in the survey, we used the listed strategies of the Expert Recommendations for Implementing Change project.^23^ The research team made a selection of the strategies that were most often used in injury prevention research and, if necessary, changes were made to the wording for the strategies to fit the injury prevention context. For the components of the process evaluation, we listed the components that are most often used in sports injury prevention based on expert opinion (ie, authors of this manuscript). The questions concerning the facilitators and barriers included prompts that were derived from the CFIR principles^15^ (more details in online supplemental file 2). Finally, the survey contained several questions on the participants’ personal characteristics. The questions were either single-choice or multiple-choice, except for the questions on facilitators and barriers for the programme delivery, use in practice, compliance or adherence, which were open-ended.

#### The semi-structured interviews

We conducted the semi-structured interviews to assess facilitators and barriers for scale-up of the intervention after the effectiveness trial. For the semi-structured interviews, we used a standardised interview guide (see online supplemental file 3). This guide was developed by the research team and is based on the model of Simmons and Schiffman,^19^ who described the process of scaling up as an interaction of five elements, consisting of the innovation, the resource organisation/team, the user organisation and scaling up strategy and the environment.^19^ The interview guide contained the following domains: undertaken scale-up activities, funding, implementation strategies during scale-up, important actors, impacts and outcomes, facilitators and barriers for scale-up and future expansion plans. The interviews were conducted online using Microsoft Teams and were conducted by two researchers (RN in combination with either EV or FvN), and audio recordings were made. All participants provided verbal consent to participate in the interviews.

#### Data analysis

We present the quantitative results from the data extraction and the survey as frequencies and percentages. We transcribed the interview recordings verbatim. The extracted facilitators and barriers from the included papers, the survey’s open questions and the transcripts were coded using a codebook. Although the CFIR principles served as the foundation for the survey, we chose to apply a single, unified codebook to all qualitative data on facilitators and barriers to implementation during scale-up. This approach was intended to enhance comparability across the two phases of the study. We employed a thematic analysis based on the domains defined by Simmons and Schiffman.^19^ Three researchers were involved in developing the codebook (JN, RN, FvN). Each researcher coded a separate interview using open codes. The open codes were grouped within the themes of Simmons’ Framework, and codes were defined based on consensus among the three researchers, which resulted in a codebook. The coding of the extracted information and the open answers in the survey was conducted by one researcher (JN), and the coding of the interviews was conducted by two researchers (JN and RN). The results will be presented in four separate sections: the strategies for implementation and scale-up; the process evaluation outcome measures; the facilitators and barriers during implementation; and the facilitators and barriers during scale-up (see also table 1).

## Results

### Survey process

**Figure 1:**
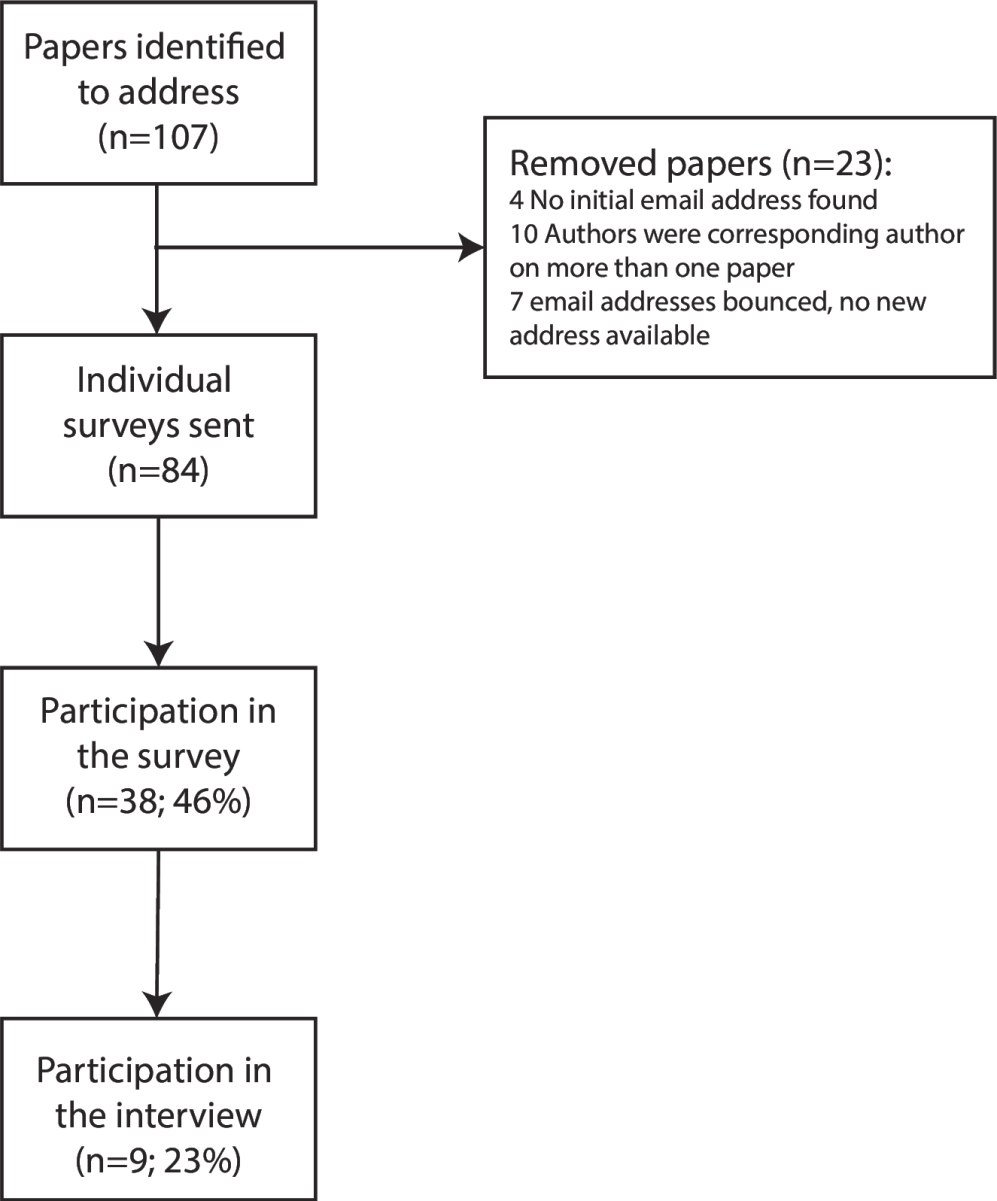
Flow chart of the study.

We extracted author information from 107 papers. Because of missing contact details (n=4), authors with multiple programmes (n=10) and authors without a working email address (n=7), 84 authors were contacted by email to complete the survey (see figure 1). 39 authors completed the survey (46%), of which 16 gave consent for a follow-up interview. Nine authors responded to the invitation to participate in the interview.

#### Characteristics of included papers and participants

All characteristics of the included studies and respondents to the survey are presented in online supplemental file 4. The majority of the included studies were randomised controlled trials (64%), included adult participants (47%) and were focused on team sports (85%). The majority of the respondents to the survey were working in Europe (56%) or North America (23%) and were working in academia (72%). During the trial, most of the respondents were principal investigators (67%), project managers (21%) and/or PhD students (33%). Of the nine interviewees, eight reported that their programme was implemented within a real-life setting, and five reported that their programme was scaled up (more details in table 2).

**Table 2:** Characteristics of interviewed participants

	Interviewee (9) N (%)
Gender (% female)	1 (11%)
Target group programme	
Adolescents (16–20 years)	2 (22%)
Adults (18+ years)	4 (44%)
Mixed ages	3 (33%)
Trial effective in reducing sports injury risk (% yes)	5 (56%)
Self-reported scale-up (% yes)	5 (56%)

#### Materials and activities employed for implementation and scale-up

Both data extraction and survey results were used to identify materials and activities employed during the trial to improve the delivery (see table 3) and during scale-up, 14 respondents reported that their programme was scaled up (36%). Of the 19 potential implementation strategies asked for in the questionnaire, 9 were reported on in the included papers. However, in the survey, the authors reported using many more strategies. The implementation strategies that were reported most, both in the survey and the included papers, were ‘In-person training and education of staff’ (papers: 43%, survey implementation: 79%, survey scale-up: 64%) and ‘Providing of supportive materials’ (papers: 39%, survey implementation: 64%, survey scale-up: 57%).

**Table 3:** Materials and activities employed to improve the use of the programme during the trial and scale-up as reported in the selected papers and the survey

Materials and activities	Implementation during trial		During scale-up
	Strategies described in selected papers (n=107)	Strategies reported in the survey (n=39)	Strategies described in the survey (n=14)
In-person/face-to-face training and education of staff (eg, coach, sports trainer, sports physician)	46 (43%)	31 (79%)	9 (64%)
Supportive materials (handouts, instruction cards, etc)	42 (39%)	25 (64%)	8 (57%)
Manual with instructions for programme delivery/use in practice (either electronic or on paper)	–	25 (64%)	6 (43%)
Providing assistance by the research team	6 (6%)	18 (46%)	3 (21%)
Endorsement from sports governing bodies, leagues, clubs, etc	–	17 (44%)	10 (71%)
Supervision visits	6 (6%)	14 (36%)	1 (7%)
Public presentation or invited lecture (eg, for clubs or coaches)	–	11 (28%)	6 (43%)
Feedback on implementation	–	10 (26%)	1 (7%)
Website with information	3 (3%)	10 (26%)	9 (64%)
Online video with instructions	–	8 (21%)	7 (50%)
Role models	2 (2%)	8 (21%)	5 (36%)
Support team	–	8 (21%)	1 (7%)
Promotional posters	–	7 (18%)	5 (36%)
Audit of implementation (quality monitoring)	–	6 (15%)	1 (7%)
Regional or nationwide (PR) campaign	–	2 (5%)	6 (43%)
Financial incentives	–	2 (5%)	–
Workshop	6 (6%)	1 (3%)	–
Newsletters	2 (2%)	–	–
Training equipment (eg, wobble boards, mats, etc)	6 (6%)	–	–
Not specified	25 (23%)	–	–
Other	7 (7%)	4 (10%)	–

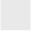
 denotes <50% of cases; 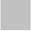 denotes 51%–75% of cases; 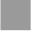 denotes >76% of cases.

#### Gathered data for process evaluation

The process evaluation methods used were derived from the selected papers and the survey. Process evaluation data were under-reported in the papers that were included in the review. In the survey, a substantial number of respondents reported having compiled compliance information (82%), exposure (82%), number of people included (67%), adherence (62%), participant enrolment (62%) and programme dose (56%). Details are provided in table 4.

**Table 4:** Process evaluation methods used

	Process evaluation measures described in selected papers (n=107) N yes (%)	Process evaluation measures reported in the survey (n=39) N yes (%)
Compliance	17 (16%)	32 (82%)
Exposure	13 (12%)	32 (82%)
Number of people (intermediaries) included/trained	0 (0%)	26 (67%)
Adherence	5 (5%)	24 (62%)
Participant enrolment	0 (0%)	24 (62%)
Programme dose	2 (2%)	22 (56%)
User satisfaction	3 (3%)	16 (41%)
Programme costs	2 (2%)	8 (21%)
Programme fidelity	1 (1%)	7 (18%)
Other	–	1 (3%)

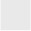
denotes <50% of cases, 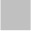 denotes 51%–75% of cases, 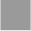 denotes 51%–75% of cases.

#### Facilitators and barriers to implementation during the trial

19 survey respondents (49%) reported formally assessing facilitators and barriers to using the programme during the trial. In most cases, this was done by questionnaires, but this was not mentioned in the method section of the trial papers. Seven survey respondents (37%) indicated that facilitators and barriers were reported in the original paper, while nine (47%) reported them in another paper, which was either published or under preparation.

The facilitators and barriers to implementing the programme were reported in both the survey and the included papers. We coded them using the codebook. We provide an overview of the facilitators and barriers to the programme’s implementation as reported in the included papers and the survey in table 5. The strategies, which are one of the domains described by Simmon and Schiffman,^19^ have been described above.

**Table 5:** Facilitators and barriers to implementation of injury prevention programmes during an effectiveness trial and scale-up

Theme 1: The programme	Factors reported for implementation during the trial		Factors for scale-up
	Described in selected papers (n=107**)**	Reported in survey (n=39**)**	Described in the Interviews (n=9)
+Clearly defined target population	√	√	X
+Support system	√	√	√
+Systematic intervention development	√	√	X
±Contextually of intervention	√	√	√
±Instruction/training for intervention use	√	√	X
±Effectiveness intervention	√	√	√
±Set up intervention	√	√	√
±Timing of intervention	√	√	X
+Sports-specific intervention	√	√	√
**Theme 2: The resource team**			
+Commitment for scale-up	X	X	√
−Small resource team	X	X	√
±Involvement researcher	√	√	√
±Sufficient funding for training	X	X	√
**Theme 3: The Implementer**			
−Fitness level coach	X	√	X
−Time	√	√	X
−Limited staff	√	X	X
±Adherence to programme	√	√	X
±Experience level coach	√	√	√
±Feedback from intervention users	X	X	√
±Motivation coach and athlete	√	√	√
**Theme 4: The broader context**			
+Push by a public event	X	X	√
±Financial support	√	√	X
±Support association	√	√	√
±Support within club	X	√	X
**Theme 5: Scale-up strategies**			
+Company-owned programme	X	√	X
+Promote intervention	X	√	√
+Quality control	X	X	√

+=facilitator, −=barrier, ±=both facilitator and barrier, √=reported, X=not reported.

#### The programme

Many of the facilitators and barriers mentioned in the papers involved the content of the injury prevention programme. Facilitating factors included: short duration and easy set-up of the programme, clear and preferably free instruction materials (both digital and physical) and flexibility within the programme. An example by Gilchrist *et al*: ‘Teams were provided with replacement exercise to help alleviate boredom with the program’.^24^ The implementation was facilitated if the programme was sports-specific and had an actual training component. The perceived effectiveness of the programme was considered a facilitator. However, if physical complaints like delayed onset muscle soreness were expected, this was sometimes a barrier to conducting the programme.

The mentioned barriers included exercises that were too complex or considered boring by the athletes. The timing of the programme was also mentioned to have an impact on the programme’s implementation: ‘Access to players for recruitment in our study was not permitted until 1 week before the start of basketball season when teams were formed. As such, players were commencing the training program just before the first game of the basketball season, not during a preseason period’.^25^

Some survey respondents mentioned that the programme’s implementation was facilitated by instruction or training to explain the programme. It would have been helpful if a peer or a healthcare specialist had provided this instruction. The impact of the instruction did, however, depend on the quality of the instructor. Lastly, to fit the specific sports context, some respondents reported using terms like ‘rest’ or ‘performance enhancement’ instead of ‘injury prevention’.

The fit of the programme and its context was reported to be a facilitator. Examples included availability of materials and space, the playing level and existing routines. As Aerts *et al* explained: ‘The coaches found the jump-landing program to be easily compatible with their training and worth the invested time’.^26^ If a programme did not match the local context, respondents often reported it as a barrier to implementation. To ensure a good fit with the local context, respondents mentioned a systematic programme development that involved many stakeholders and included community stakeholders as facilitators.

#### The research team (resource team)

Many of the respondents recognised the potential impact of the involvement of a researcher on the implementation processes. This could include the service made available through participation in a study, such as free injury registration and treatment, which facilitated implementation. However, the regular check-ins by the research team through reminders and site visits were also considered to facilitate adoption and, thereby, the programme’s implementation. On the other hand, a lack of communication between the research staff and the participants was considered a barrier to implementation because of the restricted supervision. Some authors reported disagreements between participants and the research staff. An example that participation in a study may interfere with implementation was provided by Soligard *et al*: ‘Many of the coaches [that did not participate in the study] decided that the extra work would be too time-consuming’.^27^

#### The coach and/or athletes (implementer)

Many respondents reported time constraints as a barrier for coaches and athletes to perform the programme as intended. The other often-mentioned factor was the level of motivation. If the coach and/or players are not convinced of the added value of injury prevention, this was reported as a barrier to implementation: ‘[the players and coaches were] just accommodating us because they respect us but not fully bought in on results’ (respondent survey). Motivated coaches and athletes are more likely to participate in the programme, but many authors reported a reduction in motivation to participate in the programme over time.

Adherence to the programme by the athletes was positively impacted by supervision during the exercises, and having a limited number of staff members was reported as a barrier to the programme’s implementation. The authors mentioned that peer role models had a positive impact on implementation. Still, if peer role models did not take the programme seriously, this was reported to be a barrier to implementation. Bad weather was another barrier to implementation.

The personal characteristics of the trainer could also impact the programme’s implementation. One respondent specifically reported: ‘Less fit Coaches did not demonstrate some of the more challenging exercises and left them out of the warm-up’. The experience level of the coach was reported as both a facilitator and a barrier for implementation, for example, because of limited knowledge of the importance of sports injury prevention and the differences in needs by coaches: ‘inexperienced coaches usually want to have a simple structured warm-up with same exercises—more experienced coaches may want to have dozens of different variations of exercises, and they plan different warm-ups for each practice session’ (respondent survey). Also, coaches’ lack of motivation to change existing routines was considered a barrier to implementing a programme.

#### The broader context

Financial support was considered both a facilitator and a barrier to the programme’s implementation during the trial and the possibility of scale-up of the programme by respondents to the review. Support to implement an injury programme from within the club or even broader, from a governing body, was perceived as a facilitator for implementation.

#### Facilitators and barriers for scaling up a programme

The factors associated with the scaling-up of programmes were mainly based on the semi-structured interviews. Some scale-up strategies were mentioned in the survey.

#### The programme

A standalone programme, including a digital platform with the programme’s content, was considered a facilitator for scale-up. However, little things like a registration procedure to access the programme content were reported to hamper scale-up.

A simple set-up of the programme, with flexibility for the coaches to make adjustments and increase/decrease the difficulty of the exercises, was considered a facilitator for scale-up. A good fit of the programme with existing training routines was facilitated as well. Interviewees reported that scale-up was perceived as more successful if the programme matched the local context. This could, for example, be that materials were translated into the local language, a sports-specific language style was used, or it could mean that the programme matched the cultural context:

“I think [enforcing a training] was the best way because [people in my country] are also not the best at sticking to rules in general because things aren’t policed well in the country. So you’re used to … You know rules are there. You know you can’t drive a car drunk, but you’ll do it if you have to, and you know you’re not going to get caught. (Interview 5)

When the programme is too time-consuming, this was considered a barrier to scale-up. And for some target populations, the ‘one size fits all’ principle would not apply:

There’s no way [semi-pro players] ’d ever do it. It’s too simplistic. It doesn’t fit into their normal training programs and training practices. They already have established ways of doing things, so it’s less of a … The program may very well work for them. There’s no way they’re ever going to do it. (Interview 2)

Even though one interviewee reported that scale-up was initiated without very convincing results of the effectiveness trial, convincing effectiveness numbers were reported to be very helpful in pushing scale-up further.

#### The resource team

Many interviewees had regular contact with the programme implementation sites during the effectiveness trial. This contact stopped when the researcher’s contract expired. Having someone to actively roll out a programme could facilitate scale-up, but relying on one person also makes the scale-up vulnerable: ‘If the program manager were hit by a bus tomorrow, I would be a bit concerned about the longevity of the program. I think it would have a major dip or it would regress for a while. That’s the problem’ (Interview 5).

Some implementers need personal guidance to implement the programme in their local context. This support was considered a barrier to implementation. So, sufficient funding for training and support was considered a facilitator for scale-up.

#### The implementer

The motivation of the implementer (usually the coach) was considered key for scale-up. Very experienced coaches were considered to have difficulty changing their training routines. Those coaches involved in the development of a programme may be convinced, but ‘Lack of buy-in from coaches and coach educators not originally involved in research’ (respondent) was considered a barrier by some. To motivate coaches, it was deemed necessary to align messages to fit the target group’s needs. ‘You have to understand, the coaches are there to coach. They’re not there to prevent injuries. They’re not judged on how many ACLs they prevented or how many ankle sprains they prevented. They're judged on wins and losses at all levels’ (Interview 7). The motivation of coaches and athletes was also highlighted as being improved by ensuring that respected individuals within the local context stimulate the use of the programme. Giving end-users the possibility to provide feedback on the programme and its delivery during scale-up enables the programme owners to make amendments to the programme, which facilitates scale-up.

#### The broader context

A very serious injury, for example, a schoolboy who ends up in a wheelchair, can give a push to public awareness towards the importance of sports injury prevention, as expressed by one of the interviewees. A specific sports injury that was reported to be readily picked up in the media was concussions, which provided a window of opportunity to push the prevention of concussion programmes forward. But lack of ‘hotness’ may also negatively impact the awareness of other sports injuries: ‘So I’d tell people, ‘If you’re worried about your kid being able to play basketball through the entire season or soccer season or hockey season, preventing ankle problems is a bigger issue than preventing a head injury.’ That’s not the headlines’ (Interview 7). A programme also needed to fit within local legislation: a number of the interviewees reported privacy issues during scale-up, for example, because of restricted use of videos or because of the use of personal data.

Having the support of national organisations, such as the sports association or governmental consumer safety institution, was considered a major facilitator by many of the interviewees. Having those parties on board throughout the development phase keeps them involved: [the stakeholders from the sports foundation] are one of the founding fathers of the program. And I think that is important. Instead of developing something and then going to them and saying, “Hey, I’ve got something that is amazing for you.” They need to have ownership (Interview 1).

#### Scale-up strategies

To promote a programme, suggested strategies include motivating coaches through training and adding enforced quality control on specific parts of the programme. An update on the layout of the programme was also considered a valuable scale-up strategy: ‘And then the [association] took it away to their marketing department, and they made it look pretty, and they jazzed it up and put branding all over it’ (Interview 2). Having an association or company involved in the scale-up was also mentioned as a facilitator in the survey, as they can help with the branding of a programme and the promotion in a broader setting.

## Discussion

In this study, we aimed to better understand implementation strategies, facilitators and barriers to implementing sports injury prevention programmes during the effectiveness trial and beyond during scale-up. Therefore, we reached out to the authors of studies on injury prevention programmes for more information. Our study shows that although some strategies were already in place during the effectiveness trial, they were not reported in the included papers. Also, the results of a process evaluation are often not described. According to our survey results, only one in three studies resulted in some form of scale-up after the trial ended. Some of the reported facilitators and barriers apply to both implementation during the trial and scale-up (eg, contextuality and set-up of the programme, motivation of coach and athletes and support of the association). At the same time, others were only reported to impact implementation during the trial (systematic trial development, involvement of researchers) or during scale-up (a small resource team, push by a public event).

One of the main findings of the present study is that a structured description of the strategies that have been used during the trial and beyond is often very limited. Only nine types of strategies were reported in the papers, while from the survey, it became clear that researchers put many more strategies in place to support implementation. It might be that researchers are not aware that the actions they perceive as required for the data collection of a study, such as reminders or site visits, are relevant strategies and should be replicated when the programme is being implemented beyond a research setting. Another reason strategies are not extensively mentioned may be that journals typically have a word count limit, and that reporting on other aspects of the study is deemed more valuable. However, during the interviews, it was mentioned that the lack of funding and capacity to sustain these strategies hinders the actual implementation of programmes in practice. The field of sports injury prevention should be made more aware of the importance of identifying and reporting on implementation strategies.^28^

Although many survey respondents reported that process data were collected, a detailed presentation of the process evaluation results was limited. In the survey, the authors mentioned monitoring process indicators, such as compliance, the number of people (intermediaries) included/trained and end-user satisfaction. Although it may not yet be common in injury prevention, it is essential to conduct a structured process evaluation alongside each effectiveness trial, a so-called hybrid trial.^29^ Process evaluations can provide insight into the context, mechanisms and implementation processes to inform adaptations after an evaluation and improve the programme’s fit.^30^ Reporting guidelines, such as those proposed recently by van Nassau *et al*, should be incorporated in future research to ensure that the implementation process is properly assessed and that outcomes may be translated to other settings.^31^

Our findings suggest a difference in the factors that impact the implementation of injury prevention programmes during a trial and those that become important during scale-up. The implementation during a trial is more heavily impacted by factors directly related to the programme itself and the individual who delivers it. In contrast, during scale-up, the role of the resource team grows, and a shift is needed from local stakeholders (the club/the coach) to overarching governing bodies. Injury prevention requires navigating socio-technical challenges, including motivational demands on coaches and athletes and integration into complex and time-constrained training workflows.^8^ More research may be needed to increase our understanding of the factors that can drive the scale-up of injury prevention programmes. A human-centred systems approach, such as the Systems Engineering Initiative for Patient Safety, may provide a useful lens for this perspective.^32^ Studies on the successful scale-up of physical activity and nutrition programmes have already pointed out that the processes behind successful scale-up are complex and non-linear.^33^ To better understand the processes behind scale-up, it is essential that scale-up processes are structurally reflected on and that key aspects of the process are well documented.^20^

### Implications for research

It may be time for injury prevention research to move beyond the traditional sequence of prevention^5^ and the Translating Research into Injury Prevention Practice (TRIPP) framework.^9^ While these models have provided valuable guidance in describing programme effectiveness and implementation, they do not sufficiently address the practical realities of scaling up interventions into complex, real-world settings. Future injury prevention programmes and effectiveness trials should therefore be designed with a clear and explicit focus on implementability; that is, the extent to which interventions can be feasibly integrated into existing workflows, adapted to diverse contexts and sustained over time.

Although scale-up processes are non-linear, lessons have been learnt from previous experiences that led to the development of an assessment tool for policymakers and practitioners. Although effective in a specific setting, some interventions may not be suitable for scale-up. In the Intervention Scalability Assessment Tool, the context of a programme and the implementation planning are systematically described and scored to gain a more complete picture of the scalability.^34^ Researchers may also use this to assess the readiness for scale-up of their interventions and to assess potential gaps in their study design.

### Strengths and limitations

This study combined many data types, including those reported in the original papers, survey data and semi-structured interviews. This provides a broad perspective on implementation efforts during the effectiveness trials and beyond. This approach, however, has its limitations. 46% of the authors responded to our survey; they may have had more experience and interest in this research field. Selective responding is a common problem in web-based surveys.^35^ We cannot completely rule this out, but since we have 25 respondents who report that their programme was not scaled up, we assume that we were able to recruit a broad sample of experiences with effectiveness trials and scale-up. Another limitation is that our results may only apply to high-income countries since most of the published papers in injury prevention research have been conducted in these countries.

## Conclusion

Prevention programmes need to be successfully implemented and scaled up beyond their research setting to achieve widespread prevention of sports-related injuries. To make scale-up efforts more successful, factors for implementation and scale-up must be integrated into the developmental process of injury prevention programmes from the outset. Structured assessment of implementation and scale-up processes and reporting on the results are essential to move the field forward.

## Supplementary material

10.1136/bmjsem-2025-002931 online supplemental file 1

10.1136/bmjsem-2025-002931 online supplemental file 2

10.1136/bmjsem-2025-002931 online supplemental file 3

10.1136/bmjsem-2025-002931 online supplemental file 4

## Data Availability

Data are available upon reasonable request.
