# Supplementary material for: Implementing sports injury prevention programmes during and beyond effectiveness trials: a mixed methodologies study
Source: BMJ Open Sport Exerc Med. 2026 Feb 20;12(1):e002931. doi: 10.1136/bmjsem-2025-002931 (PMC12927292; doi:10.1136/bmjsem-2025-002931)
Supplement: online supplemental file 1 [file bmjsem-12-1-s001.doc]

**Appendix 1: Flow chart update Haddon review Vriend et al (2017)**

Records identified through database searching
(n = 7,980)

Records identified through other sources (n = 11)

Duplicates excluded
(n = 3,670)

Records screened based on Title & Abstract
(n = 4,310)

Abstracts excluded
(n = 4,230)

Full-text records assessed for eligibility
(n = 91)

Full-text articles excluded, reasons (n = 45):

3 No full text

12 No original research

3 Overuse injuries only

6 Wrong study design

4 Wrong outcome measure

2 No English language

4 Wrong study population

2 Duplicate

2 No research question on

preventive effect of an

intervention

1 Multiple publications on

same study

6 Duplicates with previous

review (Vriend et al., 2017)

Studies included in qualitative synthesis
(n = 37)

Total overview included papers
(n = 192)

Search Vriend 2017
(n = 155)

Studies on exercise based injury prevention
(n = 107)
