# Supplementary material for: Implementing sports injury prevention programmes during and beyond effectiveness trials: a mixed methodologies study
Source: BMJ Open Sport Exerc Med. 2026 Feb 20;12(1):e002931. doi: 10.1136/bmjsem-2025-002931 (PMC12927292; doi:10.1136/bmjsem-2025-002931)
Supplement: online supplemental file 2 [file bmjsem-12-1-s002.docx]

## Survey

**Plain Language Statement**You are invited to take part in this research project as you are the primary author or co-author of a published sport injury prevention effectiveness study. The aim of this project is to gain an overview of the impact of intervention strategies for injury prevention in daily practice after the initial trial was conducted. Therefore, we ask you to take part in this **very short survey**. It should take **less than 15 minutes** to complete and **your name is not required**. There are no anticipated risks from participation.

**Confidentiality**All data collected will be coded to protect your privacy, held in confidence and stored on a secure, password-protected server at Amsterdam UMC, location VUmc with identification numbers only. Data from the online survey are stored in a secure data centre in Switzerland and will be permanently deleted once data collection is complete and all data have been downloaded by the researchers. The online survey does not request information that allows individuals to be identified.
Only the Amsterdam UMC, location VUmc research team involved in this study will have access to your data. You are under no obligation to participate in this research and you have the right to withdraw from the survey at any point by exiting or closing the screen. **Please only complete this survey once**.

**Procedure**Please read the questions carefully and answer as honestly as possible by selecting the most suitable option. Where you are asked to write an answer, please do so the best you can in the space provided.
1. By clicking ‘**I Agree**’ below you are giving consent for your answers to be used as part of this research.
2. You are also confirming that you have read and understood the Plain Language Statement above and have had the opportunity to ask further questions or seek clarification about this research.
3. The researchers have agreed not to reveal your identity and personal details, including where information about this project is published, or presented in any public form.
4. The researchers will inform you about any future publications related to this research.

If you require further information or if you have any questions or problems concerning this project, please contact Dr. Femke van Nassau at f.vannassau@amsterdamumc.nl or Jelena Haugg at j.haugg@amsterdamumc.nl

If you wish to take part in this survey please click **'I Agree'** to begin. q0

- I Agree

Part 1
This section focuses on the process during the trial. We are interested in any activities done during the trial that may have improved intervention delivery, use in practice, compliance and adherence to the evaluated intervention.

**What materials or activities were used during the trial to improve the delivery, use in practice, compliance and/or adherence of the intervention under study?** q1

You can tick multiple answers.

- In person/ face-to-face training and education of staff (e.g. coach, sports trainer, sport physician)
- Online video with instructions
- Manual with instructions for program delivery/use in practice (either electronic or on paper)
- Supportive materials such as handouts, instruction cards
- Website with information
- Promotional posters
- Financial incentives
- Feedback on implementation
- Providing assistance
- Audit of implementation (quality monitoring)
- Role models
- Public presentation or invited lecture (e.g. for clubs or coaches)
- Endorsement from sport governing bodies, leagues, clubs, etc.
- Regional or nationwide (PR) campaign
- Supervision visits
- Support team
- No such materials and activities used
- Other

*Answer this question, when* ***“Other”*** *was selected in q1. If not continue to the* ***next question****.***Please specify what other materials or activities were used during the trial to improve the delivery, use in practice, compliance and/or adherence of the intervention under study?** q1a

Please enter your answer here:

**Did you monitor one of the following aspects during the trial?**  q2
*You can tick multiple answers.*

- Compliance
- Adherence
- Exposure
- Program dose
- Program fidelity
- User satisfaction
- Intervention costs
- Number of people (intermediaries) included/trained
- Participant enrolment
- No such data collected
- Other

*Answer this question, when “****Other****” was selected in q2. If not continue to the* ***next question****.***Please specify what other aspects you monitored during the trial related to the delivery, use in practice, compliance and/or adherence of the intervention under study?** q2a

Please enter your answer here:

**Facilitators and Barriers**

The next questions refer to facilitators and barriers for the intervention delivery, use in practice, compliance or adherence to the evaluated intervention during the trial.
Think about the following facilitating or hindering factors related to the:

**- Intervention** itself, such as relative advantage and feasibility;
**- Individuals who receive the intervention** (i.e. athletes or coaches), such as their attitude and satisfaction with the intervention;
**- Individuals who implement the intervention** (i.e. trainers, coaches), such as their knowledge and beliefs about the intervention;
**- Organisational setting**, such as staff turnover and staff capacity;
**- Broader context,** such as whether the intervention fits with existing rules and legislation, politics or cultural context.

You might have formally assessed facilitators and barriers as part of your trial, or you might have „observed“ them and kept notes on the things you observed or found did/did not work as you ran the trial, so these might be in the form of field notes or similar.

**Did you formally assess any facilitators and/or barriers for the use of the intervention in practice as part of your trial?** q3

- No
- Yes

*Answer this question only, when* ***q3*** *was selected with “****yes****”. If not continue to* ***q5.***
**Please specify how you assessed the facilitators and barriers for use in practice (e.g through questionnaires, interviews or field notes).** q3a

Please enter your answer here:

*Answer this question, when* ***q3*** *was selected with “****yes****”. If not continue to* ***q5****.***Were these facilitators and barriers reported?** q4

- No, facilitators and barriers were not reported
- Yes, facilitators and barriers were directly reported in the original trial paper
- Yes, facilitators and barriers were indirectly reported as part of the discussion in the original trial paper
- Yes, facilitators and barriers were reported in another paper/publication/report

*Answer this question, when* ***q4*** *was selected with “****Yes, facilitators and barriers were reported in another paper/publication/report****”. If not continue to* ***q5****.***Please list the references of the other papers/publications/reports.** q4a

Please enter your answer here:

**Thinking back on the trial, which main three facilitators did you identify/observe for intervention delivery, use in practice, compliance or adherence?** q5

If you didn’t assess them formally please describe what you observed or experienced.

Please enter your answer here:

**Thinking back on the trial, which main three barriers did you identify/observe for intervention delivery, use in practice, compliance or adherence?** q6

If you didn’t assess them formally please describe what you observed or experienced.

Please enter your answer here:

Part 2
This section focuses on the process **after the trial**. We are interested in any attempts to bring your intervention to sports practice on a larger scale (i.e. scale up).

**How were the research results of your trial disseminated?** q7

You can tick multiple answers.

- Scientific publication(s)
- Lay publication(s)
- Presentation(s) at conferences
- Included in guidelines
- Included in policy
- Presentation(s) to stakeholders/end users (e.g. sport federations, trainers, physiotherapists)
- Press release, interview or news/magazine article
- Not disseminated (yet)
- Other

*Answer this question, when* ***q7*** *was selected with “Other”. If not continue to* ***q8****.***Please describe how the research results of your trial were disseminated.** q7a

Please enter your answer here:

**Was any follow-up research conducted after the trial?** q8

Think about new effectiveness trials, evaluation of translation to other setting or sport, implementation study, qualitative research, study focused on bringing the intervention to practice, etc.

You can tick multiple answers.

- No
- Yes, effectiveness trial(s)
- Yes, evaluation of translation to other setting or sport
- Yes, implementation study
- Yes, qualitative research
- Yes, study focused on bringing the intervention to practice (e.g. scale up)
- Yes, other

*Answer this question, when q8 was selected with “Yes, other”. If not continue to q9.*

**Please describe other follow-up research conducted after the trial.** q8a

Please enter your answer here:

**Was the intervention used in practice after the trial?** q9

- No
- Yes
- Unsure

**Was the intervention scaled up in practice after the evaluation?** q10

- No
- Yes

*Answer this question, when* ***q10*** *was selected with “****Yes”****. If not continue to* ***q11****.*

**Please describe where the intervention was scaled up (e.g. in how many places, countries, etc.).** q10a

Please enter your answer here:

Answer **q11-q15**, when **q9** was answered with “**yes**”,
OR **q10** was answered with “**yes**” or “**unsure”**.

If not, continue with **q16**.

**What materials or activities were used after the trial to improve the reach, delivery, use in practice, compliance and/or adherence of the intervention?** q11

You can tick multiple answers.

- In person/ face-to-face training and education of staff (e.g. coach, sports trainer, sport physician)
- Online video with instructions
- Manual with instructions for program delivery/use in practice (either electronic or on paper)
- Supportive materials such as handouts, instruction cards
- Website with information
- Promotional posters
- Financial incentives
- Feedback on implementation
- Providing assistance
- Audit of implementation (quality monitoring)
- Role models
- Public presentation or invited lecture (e.g. for clubs or coaches)
- Endorsement from sport governing bodies, leagues, clubs, etc.
- Regional or nationwide (PR) campaign
- Supervision visits
- Support team
- No such materials and activities used
- Other

*Answer this question, when* ***q11*** *was selected with “****Other”****. If not continue to* ***q12***

**Please specify what other materials or activities were used after the trial to improve the reach, delivery, use in practice, compliance and/or adherence of the intervention?** q11a

Please enter your answer here:

**Thinking about the scale up process, which main three facilitators did you identify/observe during this process?** q12
 *If you didn’t assess them formally please describe what you observed or experienced.*

Please enter your answer here:

**Thinking about the scale up process, which main three barriers did you identify/observe during this process?** q13

If you didn’t assess them formally please describe what you observed or experienced.

Please enter your answer here:

**On a scale from 1 (not successful at all) to 10 (very successful), how successful was the scale up?** q14

Click on scale to place handle.

- 1
- 2
- 3
- 4
- 5
- 6
- 7
- 8
- 9
- 10

**By estimation, how many years did it take from the start of the trial to satisfactory scale up?** q15

Please enter your answer here:

Continue here, when **q 9** was answered with “**no”**. If you answered q11-15, please skip to q17 (part 3).

**Is there a reason why no scaling up attempts were made, e.g. why the intervention stopped after the trial?** q16

You can tick multiple answers.

- Lack of time for scaling up process
- Lack of funding
- Lack of commitment
- No effectiveness of intervention
- I have no idea
- Other

*Answer this question, when* ***q16*** *was selected with “****Other”****. If not continue to* ***q17***

**Please specify other reasons for no scaling up attempts after the trial?** q16a

Please enter your answer here:

Part 3 is asking about your **personal characteristics**.

**In which country do you primarily work?** q17

Please note, if you regularly work in more than one country please select where you spend the majority of your time.

Please enter your answer here:

**Which of the following describes your gender?** q18

- Male
- Female
- Other
- I prefer not to say

**Please select your age range.** q19

- 18-24 years
- 25-29 years
- 30-34 years
- 35-40 years
- 41-45 years
- 46-50 years
- 51-55 years
- 56-60 years
- 60+ years

**In which sector do you currently work?** q20

Please select all that apply.

- University/academic position
- Sports and exercise medicine (clinical)
- Support services in sport (non-clinical)
- Non-government organization (NGO) or Non-profit organization
- International-level sports organization
- National-level sports organization
- Community-level sports organization
- Research consultancy
- Other

*Answer this question, when* ***q20*** *was selected with “****Other”****. If not continue to* ***q21***

Please specify the sector you currently work. q20a

Please enter your answer here:

**How long have you been working in this sector?** q21

If you work in more than one sector, please select where you spend the majority of your time.

- 1-5 years
- 6-10 years
- 11-15 years
- 16-20 years
- 21-25 years
- Over 25 years

**In which area do you primarily work or study?** q22

Please select all that apply.

- Sports and exercise medicine
- Public health/Population health
- Clinical health
- Elite sports
- Recreational sports
- Physical activity
- Implementation science
- Psychology
- Sociology
- Epidemiology
- Health services research
- Other

*Answer this question, when* ***q22*** *was selected with “****Other”****. If not continue to* ***q23***

**Please specify in which area you primarily work or study.** q22a

Please enter your answer here:

**What is your current position?** q23

Please select all that apply.

- Academic (research, teaching and/or lecturing)
- Practicioner (clinical)
- Consultant
- Policy maker/policy officer
- Senior manager/CEO of organisation
- Practicioner (coach or other role)
- Student (including higher degree/research)
- Industry professional
- Other

*Answer this question, when* ***q23*** *was selected with “****Other”****. If not continue to* ***q24.***

**Please specify your current position** q23a

Please enter your answer here:

**What was/is your role in the research project of the paper we are referring to?** q24

Please select all that apply.

- Principal investigator
- Project manager
- Co-investigator
- PhD student
- PhD supervisor
- Clinician
- Other

*Answer this question, when* ***q24*** *was selected with “****Other”****. If not continue to* ***q25.***

**Please specify your role in the research project of the paper we are referring to.** q24

Please enter your answer here:

**Do you have any other comments, thoughts or suggestions that you think is important to mention?** q25

Please enter your answer here:

**Would you like to be contacted by a researcher to provide additional information to this project?** q26

- Yes
- No

*Answer this question, when* ***q26*** *was selected with “****yes”****.*

Name: q27

__________

Email: q28

__________

Thank you for completing this survey, your answers were saved.
