# Supplementary material for: Implementing sports injury prevention programmes during and beyond effectiveness trials: a mixed methodologies study
Source: BMJ Open Sport Exerc Med. 2026 Feb 20;12(1):e002931. doi: 10.1136/bmjsem-2025-002931 (PMC12927292; doi:10.1136/bmjsem-2025-002931)
Supplement: online supplemental file 3 [file bmjsem-12-1-s003.docx]

## Appendix 3 **-** Interview Guide

Date for interview:_________________________________________________

Location:_________________________________________________________

Respondent(s):____________________________________________________

Interviewer:______________________________________________________

Reporter: ________________________________________________________

***Introduction***

*My name is … and I am from.... I will be conducting this interview with you. The study investigates the scale-up process of different injury prevention interventions to learn more about scale-up processes and key events that have occurred as part of the scaling-up process. With scaling up we mean: “deliberate efforts to increase the impact of successfully tested innovations to benefit more people and to foster policy and programme development on a lasting basis” In short, this means: what happened with the intervention after the effect trial?*

*You have been selected for the interview because you were an author of the effect paper and agreed to be interviewed in the survey you completed. We have reached out to you for the paper (NAME PAPER)*

*All the information you give to us today will remain strictly confidential. With your permission, we would like to record the interview so we have an accurate record of what you have said. The interview will be transcribed and any identifying information either about you or the people you mention will be disguised to preserve anonymity.*

*There are no right or wrong answers to the questions - just your perceptions and recollections of events. If you would like to stop the interview at any time for whatever reason, just let us know. You can also withdraw your information in part or in full from the study if you change your mind about participating.*

*The interview should take about 60 minutes - do you have any time constraints that we should know about before starting?*

*Is there anything you would like to ask me before we get started?*

*>> start recorder<<*

*Ask on tape if the participant agrees to the interview being recorded.*

***Introduction:***

Can you tell me a little about your background and what your function/role is or was with regard to the intervention?

*Now we will start with the intervention, how it was developed and what it contained. Then we will move to what happened after the trial.*

***Intervention development*** *(prompt: different stages of scale-up)*

- Can you describe briefly the nature of the problem that the intervention was originally designed to address?
- What aims is the intervention to achieve?
- What is the target population?
- How was the intervention developed? Any co-creation?
- Can you describe ‘Intervention X’ and its components? *(prompts: Description of the Intervention in terms of Intervention objectives, Intervention design, Underlying principles/theory, Delivery format, Components, Duration, Costs)*
- What setting is the intervention delivered?
- Was there any support for delivery during the trial (e.g. manual, training, support of researchers etc.)
- Did the intervention allow tailoring to a specific context? *(prompt: Core and adaptable elements, flexibility, adaptation)*
- Was the intervention evaluated?
- Did the intervention change over time? If so, how and why?

*** Give a summary of the information of the intervention decision for scale-up***

***Scale up***

- Can you tell me what happened to the intervention after the effect trial? *(prompt: different stages of scale-up – development, efficacy, real-world trial, scale-up, sustainability)*
- How/when did the decision to scale up Intervention X happen/come about? What factors played a role?
- Could you describe what your role was in scaling up the intervention?

***Funding*** *(prompt: different stages of scale-up)*

- How did the funding come about and was this difficult?
- Who were the important actors in this?
- Who funded the intervention?
- Who funded the organization for implementation?
- Who funded the research (evaluation activities)?

***Implementation strategies*** *(prompt: different stages of scale-up)*

- What type of support was provided for implementers?
- How was that received?
- Was there an implementation plan beforehand to disseminate the intervention?
- Was there strong support/ guidance for the implementation fidelity of the intervention during scale-up?
- Any other implementation strategies/ materials?
- Did that change over time? If so, how and why?

***Important actors*** *(prompt: different stages of scale-up)*

- Who were the important actors/ stakeholders in the scale-up process?
- Why were they important?
- Were there actors that hindered the process?
- Did that change over time? If so, how and why?

***Impact and outcomes*** *(prompt: different stages of scale-up)*

- What was the reach of the intervention? Was the reach as it was expected or lower/higher?
- What was the impact on the health of the population (effect evaluation)?
- What did the people who used this intervention think of the intervention (process evaluation)?

***Facilitators for scale-up/ cease scale-up*** *(prompt: different stages of scale-up)*

- Were there any facilitators on the intervention level, individual level, provider level, organizational level, and community level?
- Did that change over time? If so, how and why?

***Barriers for scale-up/ cease scale-up*** *(prompt: different stages of scale-up)*

- Were there any barriers on the intervention level, individual level, provider level, organizational level, and community level?
- Did that change over time? If so, how and why?

***Future***

- Are there any planned modifications or future expansion plans (ie further scale-up) for this intervention?

***Closing***

- Is there anything else you think is important that we have not talked about?
- Thank interviewee
- Explain the future steps of a research project

*>> stop recorder<<*
