# Supplementary material for: Implementing sports injury prevention programmes during and beyond effectiveness trials: a mixed methodologies study
Source: BMJ Open Sport Exerc Med. 2026 Feb 20;12(1):e002931. doi: 10.1136/bmjsem-2025-002931 (PMC12927292; doi:10.1136/bmjsem-2025-002931)
Supplement: online supplemental file 4 [file bmjsem-12-1-s004.docx]

Appendix 4: Characteristics of the included studies in the review

**Table 1: Characteristics of the study population in the studies included in the review (n = 107)**

| Characteristics | N (%) |
| --- | --- |
| Study follow-up | |
| 6 months or less | 28 (26%) |
| 7 – 12 months | 34 (32%) |
| 1 – 2 years | 26 (24%) |
| Over 2 years | 16 (15%) |
| Unspecified | 3 (3%) |
| Study design | |
| RCT | 68 (64%) |
| Pretest-posttest design (prospective) | 13 (12%) |
| Controlled trial | 15 (14%) |
| Other | 11 (10%) |
| Statistical significance reported | |
| Sign change/difference | 72 (67%) |
| No sign change/ no difference | 35 (33%) |
| Age of the study population |  |
| Youth <16 years) | 17 (16%) |
| Adolescents (16-20 years) | 19 (18%) |
| Adults (≥18 years) | 50 (47%) |
| Mixed | 19 (18% ) |
| Unspecified | 16 (2%) |
| Gender of the study population | |
| Male | 39 (36%) |
| Female | 20 (19%) |
| Mixed | 32 (30%) |
| Unknown | 16 (3%) |
| Specific sport characteristic | |
| Team sports | 81 (76%) |
| Individual sports | 12 (11%) |
| Multiple | 12 (11%) |
| Other | 2 (2%) |
| Level of sports | |
| Elite/professional | 36 (34%) |
| University/College/High School | 26 (24%) |
| Recreational/Amateur | 33 (31%) |
| Other | 9 (8%) |
| Unspecified | 5 (5%) |
| Sport population | |
| General sporting population | 91 (85%) |
| Only athletes with a previous injury | 11 (10%) |
| Athletes at risk (without previous injury) | 5 (5%) |
| Continent | |
| Africa | 3 (3%) |
| Asia | 4 (4%) |
| Australia | 9 (8%) |
| Europe | 56 (52%) |
| North America | 30 (28%) |
| Multiple | 4 (4%) |
| Not specified | 1 (1%) |

**Table 2: the characteristics of the interventions and design of the studies included in the review**

| Characteristics | N (%) |
| --- | --- |
| Intervention type |  |
| Exercise program | 75 (70%) |
| Multicomponent | 12 (11%) |
| Warm-up program | 7 (7%) |
| Education | 13 (12%) |
| Type of exercise |  |
| Strength | 52 (49%) |
| Balance / coordination | 39 (36%) |
| Plyometrics / jump training | 31 (29%) |
| Stretching | 23 (21%) |
| Agility | 19 (18%) |
| Sport specific skills / technique | 13 (12%) |
| Not specified | 8 (7%) |
| Intervention duration |  |
| 6 months or less | 50 (47%) |
| 7 – 12 months | 38 (36%) |
| 1 – 2 years | 6 (6%) |
| Over 2 years | 10 (9%) |
| Intervention delivery |  |
| Coach/Trainer (to a team) a team | 44 (41%) |
| Researcher | 26 (24%) |
| Athletic trainer/(physio)therapist | 7 (7%) |
| Multiple | 17 (16%) |
| Other | 7 (7%) |
| Not specified | 6 (6%) |
| Intervention duration |  |
| 6 months or less | 50 (47%) |
| 7-12 months | 38 (36%) |
| 1 – 2 years | 16 (15%) |
| Not specified | 3 (3%) |
| Supervision |  |
| Coach/Trainer (to a team) | 38 (36%) |
| Researcher | 17 (16%) |
| No supervision | 16 (15%) |
| Medical staff members | 10 (9%) |
| Self-executed | 11 (10%) |
| Multiple | 9 (8%) |
| Other | 3 (3%) |
| Not specified | 3 (3%) |

**Table 3: characteristics of respondents to the survey on scaling up strategies**

|  | Number of responses  (total = 39) |
| --- | --- |
| Region (1 missing) |  |
| Africa | 1 (3%) |
| Asia | 2 (5%) |
| Australia | 3 (8%) |
| Europe | 22 (56%) |
| North America | 9 (23%) |
| South America | 1 (3%) |
| Gender (female) | 11 (28%) |
| Age (years) |  |
| 30 – 40 | 14 (36%) |
| 41 – 50 | 11 (28%) |
| 51 and older | 14 (36%) |
| Years of experience (years) |  |
| 5 or less | 3 (8%) |
| 6 – 20 | 22 (56%) |
| Over 20 | 13 (33%) |
| Work section (multiple responses possible) |  |
| Academic position | 28 (72%) |
| Sport and exercise medicine (clinical) | 15 (38%) |
| Support services in sport (non-clinical) | 15 (38%) |
| Sport organization | 13 (33%) |
| Role in project of interest (multiple responses possible) |  |
| Principal investigator | 26 (67%) |
| Project manager | 8 (21%) |
| PhD student | 13 (33%) |
| Clinician | 5 (13%) |

List of studies included in the review

(1-107)

1. Achenbach L, Krutsch V, Weber J, Nerlich M, Luig P, Loose O, et al. Neuromuscular exercises prevent severe knee injury in adolescent team handball players. Knee surgery, sports traumatology, arthroscopy. 2018;26:1901-8.

2. Aerts I, Cumps E, Verhagen E, Mathieu N, Van Schuerbeeck S, Meeusen R. A 3-month jump-landing training program: a feasibility study using the RE-AIM framework. Journal of Athletic Training. 2013;48(3):296-305.

3. Al Attar WSA, Soomro N, Pappas E, Sinclair PJ, Sanders RH. Adding a post-training FIFA 11+ exercise program to the pre-training FIFA 11+ injury prevention program reduces injury rates among male amateur soccer players: a cluster-randomised trial. Journal of physiotherapy. 2017;63(4):235-42.

4. Arnason A, Andersen T, Holme I, Engebretsen L, Bahr R. Prevention of hamstring strains in elite soccer: an intervention study. Scandinavian journal of medicine & science in sports. 2008;18(1):40-8.

5. Arnason A, Engebretsen L, Bahr R. No effect of a video-based awareness program on the rate of soccer injuries. The American Journal of Sports Medicine. 2005;33(1):77-84.

6. Askling C, Karlsson J, Thorstensson A. Hamstring injury occurrence in elite soccer players after preseason strength training with eccentric overload. Scandinavian journal of medicine & science in sports. 2003;13(4):244-50.

7. Attwood MJ, Roberts SP, Trewartha G, England ME, Stokes KA. Efficacy of a movement control injury prevention programme in adult men’s community rugby union: a cluster randomised controlled trial. British journal of sports medicine. 2018;52(6):368-74.

8. Baltich J, Emery C, Whittaker J, Nigg B. Running injuries in novice runners enrolled in different training interventions: a pilot randomized controlled trial. Scandinavian Journal of Medicine & Science in Sports. 2017;27(11):1372-83.

9. Bixler B, Jones RL. High-school football injuries: effects of a post-halftime warm-up and stretching routine. Family Practice Research Journal. 1992;12(2):131-9.

10. Bonato M, Benis R, La Torre A. Neuromuscular training reduces lower limb injuries in elite female basketball players. A cluster randomized controlled trial. Scandinavian journal of medicine & science in sports. 2018;28(4):1451-60.

11. Bredeweg SW, Zijlstra S, Bessem B, Buist I. The effectiveness of a preconditioning programme on preventing running-related injuries in novice runners: a randomised controlled trial. British journal of sports medicine. 2012;46(12):865-70.

12. Brooks JH, Fuller CW, Kemp SP, Reddin DB. Incidence, risk, and prevention of hamstring muscle injuries in professional rugby union. The American journal of sports medicine. 2006;34(8):1297-306.

13. Brown J, Verhagen E, Knol D, Van Mechelen W, Lambert MI. The effectiveness of the nationwide B ok S mart rugby injury prevention program on catastrophic injury rates. Scandinavian journal of medicine & science in sports. 2016;26(2):221-5.

14. Buist I, Bredeweg SW, Van Mechelen W, Lemmink KA, Pepping G-J, Diercks RL. No effect of a graded training program on the number of running-related injuries in novice runners: a randomized controlled trial. The American journal of sports medicine. 2008;36(1):33-9.

15. Cahill BR, Griffith EH. Effect of preseason conditioning on the incidence and severity of high school football knee injuries. The American journal of sports medicine. 1978;6(4):180-4.

16. Caraffa A, Cerulli G, Projetti M, Aisa G, Rizzo A. Prevention of anterior cruciate ligament injuries in soccer: a prospective controlled study of proprioceptive training. Knee surgery, sports traumatology, arthroscopy. 1996;4(1):19-21.

17. Chaiwanichsiri D, Lorprayoon E, Noomanoch L. Star excursion balance training: effects on ankle functional stability after ankle sprain. Journal-Medical Association Of Thailand. 2005;88:S90.

18. Chan ZY, Zhang JH, Au IP, An WW, Shum GL, Ng GY, et al. Gait retraining for the reduction of injury occurrence in novice distance runners: 1-year follow-up of a randomized controlled trial. The American journal of sports medicine. 2018;46(2):388-95.

19. Clark JF, Graman P, Ellis JK, Mangine RE, Rauch JT, Bixenmann B, et al. An Exploratory Study of the Potential Effects of Vision Training on Concussion Incidence in Football. Optometry & Visual Performance. 2015;3(2).

20. Croisier J-L, Ganteaume S, Binet J, Genty M, Ferret J-M. Strength imbalances and prevention of hamstring injury in professional soccer players: a prospective study. The American journal of sports medicine. 2008;36(8):1469-75.

21. Cumps E, Verhagen E, Meeusen R. Efficacy of a sports specific balance training programme on the incidence of ankle sprains in basketball. Journal of sports science & medicine. 2007;6(2):212.

22. de Hoyo M, Pozzo M, Sañudo B, Carrasco L, Gonzalo-Skok O, Domínguez-Cobo S, et al. Effects of a 10-week in-season eccentric-overload training program on muscle-injury prevention and performance in junior elite soccer players. International journal of sports physiology and performance. 2015;10(1):46-52.

23. del Ama Espinosa G, Pöyhönen T, Aramendi JF, Samaniego JC, Emparanza Knörr JI, Kyröläinen H. Effects of an eccentric training programme on hamstring strain injuries in women football players. Biomed Hum Kinet. 2015;7(1):125-34.

24. Edvardsson A, Ivarsson A, Johnson U. Is a cognitive-behavioural biofeedback intervention useful to reduce injury risk in junior football players? Journal of sports science & medicine. 2012;11(2):331.

25. Eils E, Schroeter R, Schröder M, Gerss J, Rosenbaum D. Multistation proprioceptive exercise program prevents ankle injuries in basketball. Medicine & Science in Sports & Exercise. 2010;42(11):2098-105.

26. Emery C, Meeuwisse W. The effectiveness of a neuromuscular prevention strategy to reduce injuries in youth soccer: a cluster-randomised controlled trial. British journal of sports medicine. 2010;44(8):555-62.

27. Emery CA, Cassidy JD, Klassen TP, Rosychuk RJ, Rowe BH. Effectiveness of a home-based balance-training program in reducing sports-related injuries among healthy adolescents: a cluster randomized controlled trial. Cmaj. 2005;172(6):749-54.

28. Emery CA, Rose MS, McAllister JR, Meeuwisse WH. A prevention strategy to reduce the incidence of injury in high school basketball: a cluster randomized controlled trial. Clinical Journal of Sport Medicine. 2007;17(1):17-24.

29. Engebretsen AH, Myklebust G, Holme I, Engebretsen L, Bahr R. Prevention of injuries among male soccer players: a prospective, randomized intervention study targeting players with previous injuries or reduced function. The American journal of sports medicine. 2008;36(6):1052-60.

30. Ettlinger CF, Johnson RJ, Shealy JE. A method to help reduce the risk of serious knee sprains incurred in alpine skiing. The American journal of sports medicine. 1995;23(5):531-7.

31. Farhan AF, Stephany MJ, Mahammed SK. Prevention of Soccer-Related Ankle Injuries in Youth Amateur Players: A Randomized Controlled Trial. Malaysian Journal of Movement, Health & Exercise. 2017;6(1):39-45.

32. Finch CF, Gray SE, Akram M, Donaldson A, Lloyd DG, Cook JL. Controlled ecological evaluation of an implemented exercise-training programme to prevent lower limb injuries in sport: population-level trends in hospital-treated injuries. British journal of sports medicine. 2019;53(8):487-92.

33. Finch CF, Twomey DM, Fortington LV, Doyle TL, Elliott BC, Akram M, et al. Preventing Australian football injuries with a targeted neuromuscular control exercise programme: comparative injury rates from a training intervention delivered in a clustered randomised controlled trial. Injury prevention. 2016;22(2):123-8.

34. Foss KDB, Thomas S, Khoury JC, Myer GD, Hewett TE. A school-based neuromuscular training program and sport-related injury incidence: a prospective randomized controlled clinical trial. Journal of athletic training. 2018;53(1):20-8.

35. Fredberg U, Bolvig L, Andersen NT. Prophylactic training in asymptomatic soccer players with ultrasonographic abnormalities in Achilles and patellar tendons: the Danish Super League Study. The American journal of sports medicine. 2008;36(3):451-60.

36. Gatterer H, Ruedl G, Faulhaber M, Regele M, Burtscher M. Effects of the performance level and the FIFA “11” injury prevention program on the injury rate in Italian male amateur soccer players. J Sports Med Phys Fitness. 2012;52(1):80-4.

37. Gilchrist J, Mandelbaum BR, Melancon H, Ryan GW, Silvers HJ, Griffin LY, et al. A randomized controlled trial to prevent noncontact anterior cruciate ligament injury in female collegiate soccer players. The American journal of sports medicine. 2008;36(8):1476-83.

38. Grooms DR, Palmer T, Onate JA, Myer GD, Grindstaff T. Soccer-specific warm-up and lower extremity injury rates in collegiate male soccer players. Journal of athletic training. 2013;48(6):782-9.

39. Hammes D, Aus der Fünten K, Kaiser S, Frisen E, Bizzini M, Meyer T. Injury prevention in male veteran football players–a randomised controlled trial using “FIFA 11+”. Journal of sports sciences. 2015;33(9):873-81.

40. Harøy J, Clarsen B, Wiger EG, Øyen MG, Serner A, Thorborg K, et al. The Adductor Strengthening Programme prevents groin problems among male football players: a cluster-randomised controlled trial. British journal of sports medicine. 2019;53(3):150-7.

41. Heidt RS, Sweeterman LM, Carlonas RL, Traub JA, Tekulve FX. Avoidance of soccer injuries with preseason conditioning. The American journal of sports medicine. 2000;28(5):659-62.

42. Hejna WF, Rosenberg A, Buturusis DJ, Krieger A. The prevention of sports injuries in high school students through strength training. Strength & Conditioning Journal. 1982;4(1):28-31.

43. Hespanhol LC, van Mechelen W, Verhagen E. Effectiveness of online tailored advice to prevent running-related injuries and promote preventive behaviour in Dutch trail runners: a pragmatic randomised controlled trial. British journal of sports medicine. 2018;52(13):851-8.

44. Hewett TE, Lindenfeld TN, Riccobene JV, Noyes FR. The effect of neuromuscular training on the incidence of knee injury in female athletes. The American journal of sports medicine. 1999;27(6):699-706.

45. Hides JA, Stanton WR. Can motor control training lower the risk of injury for professional football players? Medicine & Science in Sports & Exercise. 2014;46(4):762-8.

46. Hislop MD, Stokes KA, Williams S, McKay CD, England ME, Kemp SP, et al. Reducing musculoskeletal injury and concussion risk in schoolboy rugby players with a pre-activity movement control exercise programme: a cluster randomised controlled trial. British journal of sports medicine. 2017;51(15):1140-6.

47. Holme E, Magnusson S, Becher K, Bieler T, Aagaard P, Kjaer M. The effect of supervised rehabilitation on strength, postural sway, position sense and re‐injury risk after acute ankle ligament sprain. Scandinavian journal of medicine & science in sports. 1999;9(2):104-9.

48. Hupperets MD, Verhagen EA, Van Mechelen W. Effect of unsupervised home based proprioceptive training on recurrences of ankle sprain: randomised controlled trial. Bmj. 2009;339.

49. Imai A, Imai T, Iizuka S, Kaneoka K. A trunk stabilization exercise warm-up may reduce ankle injuries in junior soccer players. International journal of sports medicine. 2018;39(04):270-4.

50. Ivarsson A, Johnson U, Andersen MB, Fallby J, Altemyr M. It pays to pay attention: A mindfulness-based program for injury prevention with soccer players. Journal of Applied Sport Psychology. 2015;27(3):319-34.

51. Jamtvedt G, Herbert RD, Flottorp S, Odgaard-Jensen J, Håvelsrud K, Barratt A, et al. A pragmatic randomised trial of stretching before and after physical activity to prevent injury and soreness. British journal of sports medicine. 2010;44(14):1002-9.

52. Johnson U, Ekengren J, Andersen MB. Injury prevention in Sweden: Helping soccer players at risk. Journal of sport and exercise psychology. 2005;27(1):32-8.

53. Jørgensen U, Fredensborg T, Haraszuk JP, Crone KL. Reduction of injuries in downhill skiing by use of an instructional ski‐video: a prospective randomised intervention study. Knee Surgery, sports traumatology, arthroscopy. 1998;6(3):194-200.

54. Junge A, Lamprecht M, Stamm H, Hasler H, Bizzini M, Tschopp M, et al. Countrywide campaign to prevent soccer injuries in Swiss amateur players. The American journal of sports medicine. 2011;39(1):57-63.

55. Junge A, Rösch D, Peterson L, Graf-Baumann T, Dvorak J. Prevention of soccer injuries: a prospective intervention study in youth amateur players. The American journal of sports medicine. 2002;30(5):652-9.

56. Kelly ST, Lodge CA. Effects of the GAA15 in reducing lower extremity injury rates in adolescent males participating in hurling. Physiotherapy Practice and Research. 2018;39(2):99-105.

57. Kerr ZY, Dalton SL, Roos KG, Djoko A, Phelps J, Dompier TP. Comparison of Indiana high school football injury rates by inclusion of the USA Football “Heads Up Football” player safety coach. Orthopaedic journal of sports medicine. 2016;4(5):2325967116648441.

58. Kolt GS, Hume PA, Smith P, Williams MM. Effects of a stress-management program on injury and stress of competitive gymnasts. Perceptual and motor skills. 2004;99(1):195-207.

59. Kraemer R, Knobloch K. A soccer-specific balance training program for hamstring muscle and patellar and achilles tendon injuries: an intervention study in premier league female soccer. The American journal of sports medicine. 2009;37(7):1384-93.

60. LaBella CR, Huxford MR, Grissom J, Kim K-Y, Peng J, Christoffel KK. Effect of neuromuscular warm-up on injuries in female soccer and basketball athletes in urban public high schools: cluster randomized controlled trial. Archives of pediatrics & adolescent medicine. 2011;165(11):1033-40.

61. Lehnhard RA, Lehnhard HR, Young R, Butterfield SA. Monitoring injuries on a college soccer team: the effect of strength training. Journal of Strength and Conditioning Research. 1996;10:115-9.

62. Longo UG, Loppini M, Berton A, Marinozzi A, Maffulli N, Denaro V. The FIFA 11+ program is effective in preventing injuries in elite male basketball players: a cluster randomized controlled trial. The American journal of sports medicine. 2012;40(5):996-1005.

63. Maddison R, Prapavessis H. A psychological approach to the prediction and prevention of athletic injury. Journal of Sport and Exercise Psychology. 2005;27(3):289-310.

64. Malliou P, Gioftsidou A, Pafis G, Beneka A, Godolias G. Proprioceptive training (balance exercises) reduces lower extremity injuries in young soccer players. Journal of back and musculoskeletal rehabilitation. 2004;17(3-4):101-4.

65. Mandelbaum BR, Silvers HJ, Watanabe DS, Knarr JF, Thomas SD, Griffin LY, et al. Effectiveness of a neuromuscular and proprioceptive training program in preventing anterior cruciate ligament injuries in female athletes: 2-year follow-up. The American journal of sports medicine. 2005;33(7):1003-10.

66. McGuine TA, Keene JS. The effect of a balance training program on the risk of ankle sprains in high school athletes. The American journal of sports medicine. 2006;34(7):1103-11.

67. McHugh MP, Tyler TF, Mirabella MR, Mullaney MJ, Nicholas SJ. The effectiveness of a balance training intervention in reducing the incidence of noncontact ankle sprains in high school football players. The American journal of sports medicine. 2007;35(8):1289-94.

68. Melegati G, Tornese D, Gevi M, Trabattoni A, Pozzi G, Schonhuber H, et al. Reducing muscle injuries and reinjuries in one Italian professional male soccer team. Muscles, ligaments and tendons journal. 2014;3(4):324.

69. Noyes FR, Barber-Westin SD. Neuromuscular retraining in female adolescent athletes: Effect on athletic performance indices and noncontact anterior cruciate ligament injury rates. Sports. 2015;3(2):56-76.

70. Olmedilla-Zafra A, Rubio VJ, Ortega E, García-Mas A. Effectiveness of a stress management pilot program aimed at reducing the incidence of sports injuries in young football (soccer) players. Physical Therapy in Sport. 2017;24:53-9.

71. Olsen O-E, Myklebust G, Engebretsen L, Holme I, Bahr R. Exercises to prevent lower limb injuries in youth sports: cluster randomised controlled trial. Bmj. 2005;330(7489):449.

72. Omi Y, Sugimoto D, Kuriyama S, Kurihara T, Miyamoto K, Yun S, et al. Effect of hip-focused injury prevention training for anterior cruciate ligament injury reduction in female basketball players: a 12-year prospective intervention study. The American journal of sports medicine. 2018;46(4):852-61.

73. Owen AL, Wong DP, Dellal A, Paul DJ, Orhant E, Collie S. Effect of an injury prevention program on muscle injuries in elite professional soccer. The Journal of Strength & Conditioning Research. 2013;27(12):3275-85.

74. Owoeye OB, Akinbo SR, Tella BA, Olawale OA. Efficacy of the FIFA 11+ warm-up programme in male youth football: a cluster randomised controlled trial. Journal of sports science & medicine. 2014;13(2):321.

75. Pasanen K, Parkkari J, Pasanen M, Hiilloskorpi H, Mäkinen T, Järvinen M, et al. Neuromuscular training and the risk of leg injuries in female floorball players: cluster randomised controlled study. Bmj. 2008;337.

76. Petersen J, Thorborg K, Nielsen MB, Budtz-Jørgensen E, Hölmich P. Preventive effect of eccentric training on acute hamstring injuries in men’s soccer: a cluster-randomized controlled trial. The American journal of sports medicine. 2011;39(11):2296-303.

77. Petersen W, Braun C, Bock W, Schmidt K, Weimann A, Drescher W, et al. A controlled prospective case control study of a prevention training program in female team handball players: the German experience. Archives of orthopaedic and trauma surgery. 2005;125:614-21.

78. Quarrie KL, Gianotti SM, Hopkins WG, Hume PA. Effect of nationwide injury prevention programme on serious spinal injuries in New Zealand rugby union: ecological study. Bmj. 2007;334(7604):1150.

79. Richmond SA, Kang J, Doyle-Baker PK, Nettel-Aguirre A, Emery CA. A school-based injury prevention program to reduce sport injury risk and improve healthy outcomes in youth: a pilot cluster-randomized controlled trial. Clinical journal of sport medicine. 2016;26(4):291-8.

80. Rössler R, Junge A, Bizzini M, Verhagen E, Chomiak J, Aus der Fünten K, et al. A multinational cluster randomised controlled trial to assess the efficacy of ‘11+ Kids’: a warm-up programme to prevent injuries in children’s football. Sports medicine. 2018;48:1493-504.

81. Scase E, Cook J, Makdissi M, Gabbe B, Shuck L. Teaching landing skills in elite junior Australian football: evaluation of an injury prevention strategy. British Journal of Sports Medicine. 2006;40(10):834-8.

82. Schlingermann BE, Lodge CA, Gissane C, Rankin PM. Effects of the Gaelic Athletic Association 15 on lower extremity injury incidence and neuromuscular functional outcomes in collegiate Gaelic games. The Journal of Strength & Conditioning Research. 2018;32(7):1993-2001.

83. Seagrave III RA, Perez L, McQueeney S, Toby EB, Key V, Nelson JD. Preventive effects of eccentric training on acute hamstring muscle injury in professional baseball. Orthopaedic journal of sports medicine. 2014;2(6):2325967114535351.

84. Sebelien C, Stiller C, Maher S, Qu X. Effects of implementing Nordic hamstring exercises for semi-professional soccer players in Akershus, Norway. Orthop Pract. 2014;26(2):90-7.

85. Silvers-Granelli H, Mandelbaum B, Adeniji O, Insler S, Bizzini M, Pohlig R, et al. Efficacy of the FIFA 11+ injury prevention program in the collegiate male soccer player. The American journal of sports medicine. 2015;43(11):2628-37.

86. Silvers-Granelli HJ, Bizzini M, Arundale A, Mandelbaum BR, Snyder-Mackler L. Does the FIFA 11+ injury prevention program reduce the incidence of ACL injury in male soccer players? Clinical Orthopaedics and Related Research®. 2017;475:2447-55.

87. Söderman K, Werner S, Pietilä T, Engström B, Alfredson H. Balance board training: prevention of traumatic injuries of the lower extremities in female soccer players? A prospective randomized intervention study. Knee surgery, sports traumatology, arthroscopy. 2000;8(6):356-63.

88. Soligard T, Myklebust G, Steffen K, Holme I, Silvers H, Bizzini M, et al. Comprehensive warm-up programme to prevent injuries in young female footballers: cluster randomised controlled trial. Bmj. 2008;337.

89. Steffen K, Meeuwisse WH, Romiti M, Kang J, McKay C, Bizzini M, et al. Evaluation of how different implementation strategies of an injury prevention programme (FIFA 11+) impact team adherence and injury risk in Canadian female youth football players: a cluster-randomised trial. British journal of sports medicine. 2013;47(8):480-7.

90. Tranaeus U, Johnson U, Engström B, Skillgate E, Werner S. A psychological injury prevention group intervention in Swedish floorball. Knee surgery, sports traumatology, arthroscopy. 2015;23:3414-20.

91. Tranaeus U, Johnson U, Ivarsson A, Engström B, Skillgate E, Werner S. Sports injury prevention in Swedish elite floorball players: evaluation of two consecutive floorball seasons. Knee Surgery, Sports Traumatology, Arthroscopy. 2015;23:899-905.

92. Tyler TF, Nicholas SJ, Campbell RJ, Donellan S, McHugh MP. The effectiveness of a preseason exercise program to prevent adductor muscle strains in professional ice hockey players. The American journal of sports medicine. 2002;30(5):680-3.

93. van Beijsterveldt AM, van de Port IG, Krist MR, Schmikli SL, Stubbe JH, Frederiks JE, et al. Effectiveness of an injury prevention programme for adult male amateur soccer players: a cluster-randomised controlled trial. British journal of sports medicine. 2012;46(16):1114-8.

94. van de Hoef PA, Brink MS, Huisstede BM, van Smeden M, de Vries N, Goedhart EA, et al. Does a bounding exercise program prevent hamstring injuries in adult male soccer players?–A cluster‐RCT. Scandinavian journal of medicine & science in sports. 2019;29(4):515-23.

95. Van der Horst N, Smits D-W, Petersen J, Goedhart EA, Backx FJ. The preventive effect of the Nordic hamstring exercise on hamstring injuries in amateur soccer players: a randomized controlled trial. The American journal of sports medicine. 2015;43(6):1316-23.

96. van Mechelen W, Hlobil H, Kemper HC, Voorn WJ, de Jongh HR. Prevention of running injuries by warm-up, cool-down, and stretching exercises. The American journal of sports medicine. 1993;21(5):711-9.

97. Verhagen E, Van Der Beek A, Twisk J, Bouter L, Bahr R, Van Mechelen W. The effect of a proprioceptive balance board training program for the prevention of ankle sprains: a prospective controlled trial. The American journal of sports medicine. 2004;32(6):1385-93.

98. Verrall GM, Slavotinek JP, Barnes P. The effect of sports specific training on reducing the incidence of hamstring injuries in professional Australian Rules football players. British journal of sports medicine. 2005;39(6):363-8.

99. Waldén M, Atroshi I, Magnusson H, Wagner P, Hägglund M. Prevention of acute knee injuries in adolescent female football players: cluster randomised controlled trial. Bmj. 2012;344.

100. Wedderkopp N, Kaltoft M, Holm R, Froberg K. Comparison of two intervention programmes in young female players in European handball–with and without ankle disc. Scandinavian journal of medicine & science in sports. 2003;13(6):371-5.

101. Wedderkopp N, Kaltoft M, Lundgaard B, Rosendahl M, Froberg K. Prevention of injuries in young female players in European team handball. A prospective intervention study. Scandinavian journal of medicine & science in sports. 1999;9(1):41-7.

102. Wester JU, Jespersen SM, Nielsen KD, Neumann L. Wobble board training after partial sprains of the lateral ligaments of the ankle: a prospective randomized study. Journal of Orthopaedic & Sports Physical Therapy. 1996;23(5):332-6.

103. Ytterstad B. The Harstad injury prevention study: the epidemiology of sports injuries. An 8 year study. British journal of sports medicine. 1996;30(1):64-8.

104. Zakaria AA, Kiningham RB, Sen A. Effects of static and dynamic stretching on injury prevention in high school soccer athletes: A randomized trial. Journal of sport rehabilitation. 2015;24(3):229-35.

105. Zouita S, Zouita AB, Kebsi W, Dupont G, Abderrahman AB, Salah FZB, et al. Strength training reduces injury rate in elite young soccer players during one season. The Journal of Strength & Conditioning Research. 2016;30(5):1295-307.

106. Kerr G, Goss J. The effects of a stress management program on injuries and stress levels. Journal of applied sport psychology. 1996;8(1):109-17.

107. Pfeiffer RP, Shea KG, Roberts D, Grandstrand S, Bond L. Lack of effect of a knee ligament injury prevention program on the incidence of noncontact anterior cruciate ligament injury. JBJS. 2006;88(8):1769-74.
